# Raman spectroscopic imaging for quantification of depth-dependent and local heterogeneities in native and engineered cartilage

**DOI:** 10.1038/s41536-018-0042-7

**Published:** 2018-02-09

**Authors:** M. B. Albro, M. S. Bergholt, J. P. St-Pierre, A. Vinals Guitart, H. M. Zlotnick, E. G. Evita, M. M. Stevens

**Affiliations:** 1 Department of Materials, Imperial College London, London, SW7 2AZ United Kingdom; 2 Department of Bioengineering, Imperial College London, London, SW7 2AZ United Kingdom; 3 Institute of Biomedical Engineering, Imperial College London, London, SW7 2AZ United Kingdom

## Abstract

Articular cartilage possesses a remarkable, mechanically-robust extracellular matrix (ECM) that is organized and distributed throughout the tissue to resist physiologic strains and provide low friction during articulation. The ability to characterize the make-up and distribution of the cartilage ECM is critical to both understand the process by which articular cartilage undergoes disease-related degeneration and to develop novel tissue repair strategies to restore tissue functionality. However, the ability to quantitatively measure the spatial distribution of cartilage ECM constituents throughout the tissue has remained a major challenge. In this experimental investigation, we assessed the analytical ability of Raman micro-spectroscopic imaging to semi-quantitatively measure the distribution of the major ECM constituents in cartilage tissues. Raman spectroscopic images were acquired of two distinct cartilage tissue types that possess large spatial ECM gradients throughout their depth: native articular cartilage explants and large engineered cartilage tissue constructs. Spectral acquisitions were processed via multivariate curve resolution to decompose the “fingerprint” range spectra (800–1800 cm^−1^) to the component spectra of GAG, collagen, and water, giving rise to the depth dependent concentration profile of each constituent throughout the tissues. These Raman spectroscopic acquired-profiles exhibited strong agreement with profiles independently acquired via direct biochemical assaying of spatial tissue sections. Further, we harness this spectroscopic technique to evaluate local heterogeneities through the depth of cartilage. This work represents a powerful analytical validation of the accuracy of Raman spectroscopic imaging measurements of the spatial distribution of biochemical components in a biological tissue and shows that it can be used as a valuable tool for quantitatively measuring the distribution and organization of ECM constituents in native and engineered cartilage tissue specimens.

## Raman spectroscopy: combined approach examines cartilage components

A combined imaging and statistical approach could lead to a better understanding of how osteoarthritis develops and to improved cartilage engineering strategies. Molly Stevens and colleagues at Imperial College London used Raman spectroscopic imaging to quantitatively measure the spatial distribution of extracellular matrix (ECM) components in natural and engineered cartilage, which varies from one part of the tissue to another. In Raman spectroscopy, photons from a laser shined on a tissue sample are reflected in varying degrees by the molecules they interact with. The team measured these reflections and distinguished them from one another using a statistical technique called multivariate curve resolution. The method successfully measured the distribution of glycosaminoglycan chains, collagen and water in the ECM of both types of cartilage. The method could help researchers learn more about tissue degenerative diseases and develop better strategies for engineering tissues for repair and replacement.

## Introduction

Articular cartilage is a unique connective tissue that serves as the bearing material at the ends of long bones, providing smooth, pain-free articulation during joint motion. This functionality is achieved through the presence of a remarkable, mechanically-robust extracellular matrix (ECM) that is structurally organized to resist physiologic strains and provide exceptionally low friction and wear during articulation. The ability to faithfully characterize the biochemical organization and distribution of the cartilage ECM is essential for two critical areas of cartilage research: (1) understanding the process by which native articular cartilage structurally breaks down during the progression of degenerative diseases, such as osteoarthritis, and (2) developing novel cartilage repair strategies to treat degeneration and restore joint functionality.

Structurally, native articular cartilage possesses a unique ECM, consisting of large dense glycosaminoglycan (GAG) chains embedded in a fibrous collagen matrix.^1^ The ECM is spatially organized on multiple length scales, giving rise to exceptional mechanical functionality in the form of resistance to compressive loads, low friction articulation, and cellular mechanosensing. For example, on the tissue level, the concentration and alignment of GAG and collagen vary greatly throughout the depth of the tissue^2^ (from the articular surface to subchondral bone), giving rise to a high degree of mechanical resistance and frictional properties.^3^ On the cellular level, articular chondrocytes are surrounded by a biochemically distinct pericellular matrix, which is responsible for transmitting physiologic mechanical loads to individual cells.^4^ Globally, the distribution of the cartilage ECM is critical for tissue maintenance and health. During the onset and progression of osteoarthritis, the organization of the cartilage ECM is disrupted, in turn compromising the mechanical functionality of the tissue.

The primary goal of cartilage tissue engineering is to generate mechanically-functional cartilage replacement tissues that can be used to clinically repair articular cartilage defects.^5–7^ Conventionally, this strategy consists of encapsulating chondrogenic cells in a polymeric scaffold in vitro, and introducing stimulatory cues to promote the deposition of cartilaginous ECM products. A major challenge in the field is the development of engineered tissues that recapitulate the ECM content and organization of native cartilage, thus ensuring sufficient mechanical functionality and increasing the likelihood of long-term survival upon implantation in the native environment. The ability to quantitatively measure the distribution and organization of ECM in engineered cartilage would serve as a highly valuable analytical tool for the assessment of tissue engineering strategies.

Over the years, a multitude of techniques have been adopted to characterize the cartilaginous ECM for both native cartilage and engineered tissues. Conventionally, characterization techniques of GAG and collagen often include biochemical assays and histological tissue staining. Interestingly, the ability to quantify the organization or distribution of matrix products in cartilage tissues has remained a challenge; in general, biochemical assays only yield the bulk concentration of matrix constituents in the tissue, while histological staining provides a qualitative representation of spatial distribution and is notably confounded by a host of sample preparation artifacts.^8^ More recently, several supplemental characterization techniques have been developed, including second harmonic generation,^9^ two-photon fluorescence,^10^ and infrared spectroscopy.^11–14^ These techniques often possess limitations in terms of spatial resolution or biochemical specificity.

Raman spectroscopy is a non-invasive, label-free imaging technique that offers a unique potential to quantify the distribution of ECM products in cartilage tissues. This spectroscopic technique produces an optical fingerprint of the biomolecular constituents of a tissue based on inelastic light scattering (the Raman effect).^15^ Importantly, the intensity of the measured Raman spectra is, in principle, linearly proportional to the concentration of molecular constituents. As such, Raman spectroscopy measurements may be able to yield a measure of the relative concentration of molecular constituents in a localized tissue region. When combined with a microscope, Raman spectral images can be acquired, providing diffraction-limited spatial information about the relative spatial distribution of biochemical constituents.^16^ Raman spectroscopic imaging offers the unique benefit of compatibility with water and, as such, can be performed on unfixed, hydrated tissue samples, thus allowing for the elimination of laborious sample preparation techniques and the avoidance of chemical fixation artifacts. Consequently, Raman spectroscopic imaging may serve as a valuable technique for performing rapid, artifact-free, high-resolution images of GAG, collagen, and water distributions in native and engineered cartilage tissues. Through the use of a range of microscope objectives, these images can, in principle, be used to quantify ECM distributions on multiple length scales.

Interestingly, while Raman spectroscopic imaging is an emerging technique for a variety of tissue types,^17,18^ it has not been benchmarked for its quantitative analytical capability. The major challenge encountered when attempting to use Raman spectral imaging to describe the molecular components of biological tissues is the complexity associated with overlapping signals. In cartilage, the ECM is strongly dominated by collagen signals whereas the GAGs are buried and difficult to resolve. Various multivariate analyses have been applied in Raman spectroscopy to deconvolve the spectra, such as principal component analysis.^18^ However, this technique is not able to provide specific and quantifiable biochemical information. For this reason, there is a fundamental need to introduce multivariate statistical techniques that can provide intuitive biochemical interpretations. One such technique is multivariate curve resolution (MCR).^19^ Briefly, MCR deconvolves spectra based on biochemical variance by using an alternating least squares algorithm.^19^ We have recently explored the utilization of this technique to reveal complexity in the organization of native cartilage^20^ and the elaboration of the bulk matrix content in growing engineered cartilage tissues.^21^ However, the ability of this technique to quantify the distribution of ECM constituents in cartilaginous tissues remains unclear.

The aim of this investigation is to assess the ability of Raman spectroscopic imaging in conjunction with MCR to quantitatively measure the concentration distribution of cartilage ECM constituents (GAG, collagen, and water) in native and engineered cartilage tissues. Validations are employed through the implementation of an analytical technique, whereby the spatial distribution of ECM constituents in tissues is directly measured via biochemical assay analysis and statistically compared to Raman spectroscopic imaging measurements. Validations are performed on three distinct cartilage tissue model systems that possess unique spatial gradients of ECM constituent concentrations: (1) Native articular cartilage explants, (2) Engineered cartilage tissue constructs, and (3) Enzymatically treated cartilage explants. Further, we analyze the ability of Raman spectroscopic imaging to perform characterizations on tissue level heterogeneities in cartilage (throughout the entire depth of tissue), as well as localized heterogeneities.

## Results

**Fig. 1:**
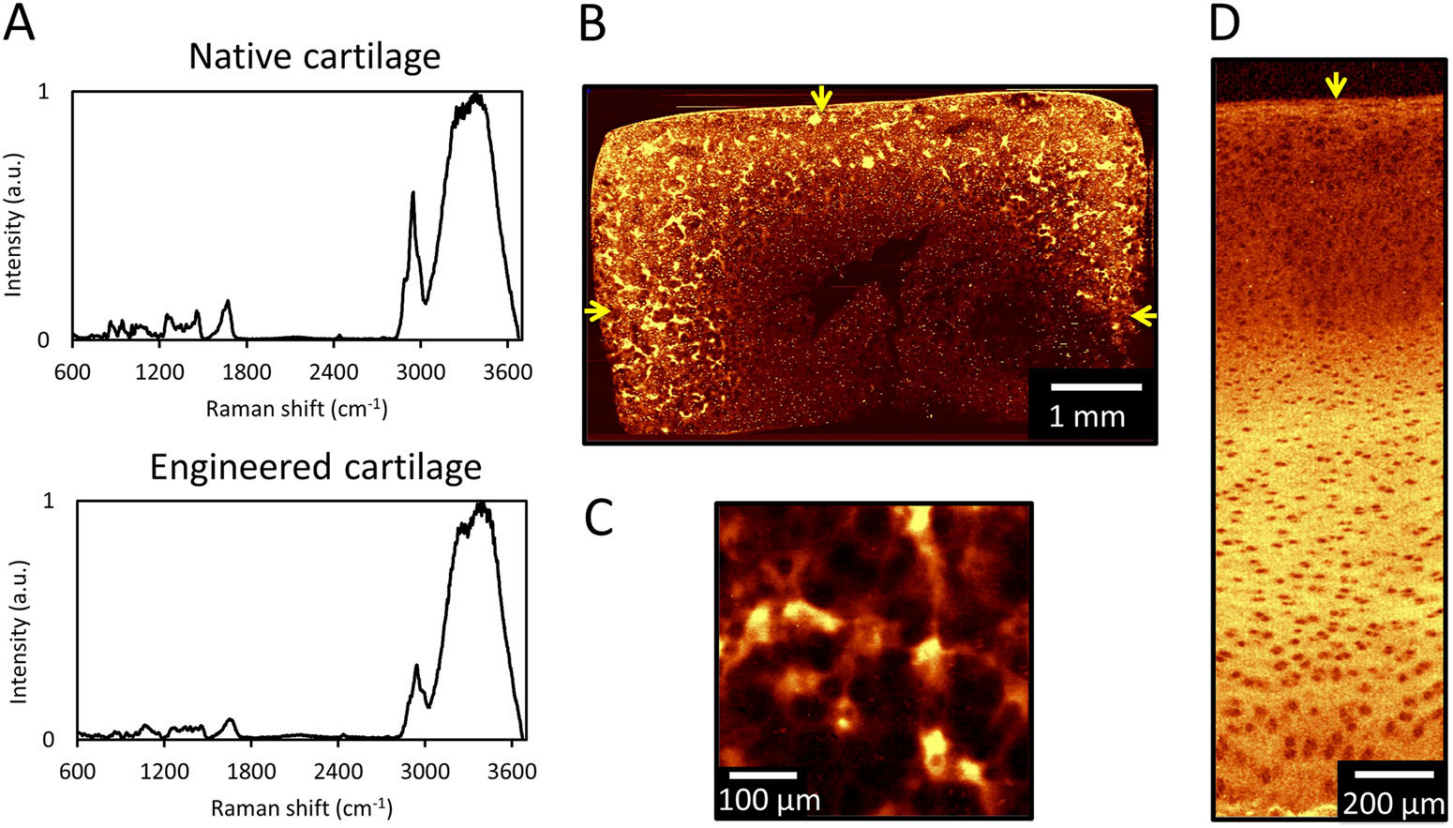
(**a**) Raw spectra of native and engineered cartilage. Raman spectroscopic image (univariate analysis) of ECM heterogeneities in 56-day cultured large engineered cartilage tissue construct (∅6 mm × 3 mm) for (**b**) the full cross-section (10 μm spatial resolution) and (**c**) a localized peripheral region (1 μm resolution). Arrows represent media-exposed surfaces. (**d**) Raman spectroscopic image of natural depth dependent ECM heterogeneities in native articular cartilage (10 μm resolution). Arrow represents articular surface

Raman spectroscopy images were measured for native (*n* = 8 tissues) and engineered cartilage (*n* = 4 tissues) (Fig. 1a). In agreement with previous studies,^22–24^ intense Raman peaks were present near 836 cm^−1^ (proline), 875 cm^−1^ (hydroxyproline), 1004 cm^−1^ (*ν*_s_(C–C) of phenylalanine), 1061 *ν*_s_(S=O) of GAGs, 1245 cm^−1^ (amide III *ν* (C–N) and *δ* (N–H) of proteins), 1450 cm^−1^ (*δ* (CH_2_) deformation of collagen) and 1672 cm^−1^ (amide I *ν* (C=O)). Since collagen is the most abundant protein in articular cartilage, univariate imaging of the protein peak (2940 cm^−1^) of engineered and native articular cartilage shows the collagen distribution in both the engineered and native tissues (Fig. 1b–d).

**Fig. 2:**
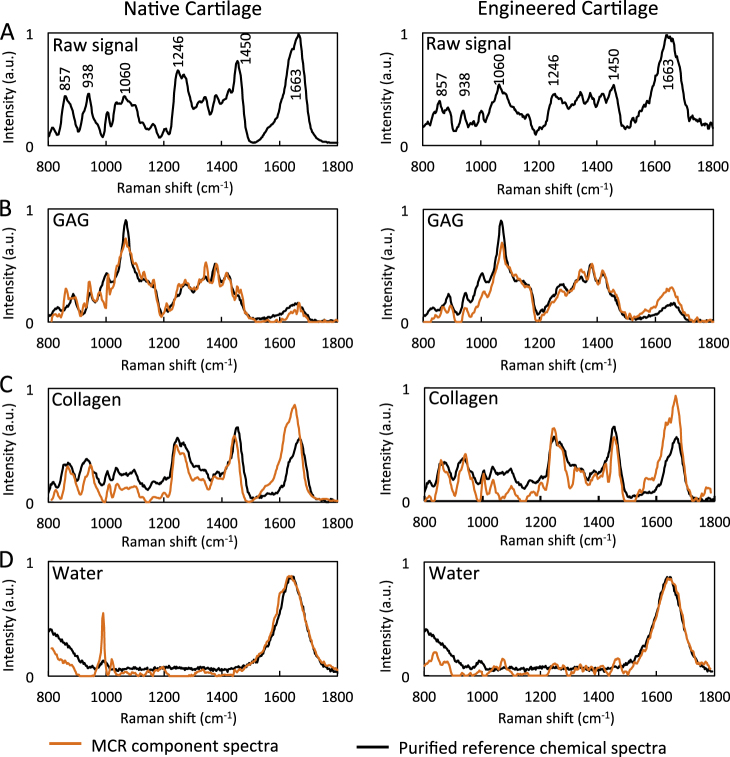
(**a**) Raw spectra of native and engineered cartilage. Component spectra of molecular constituents (**b**) GAG, (**c**) collagen, (**d**) water following deconvolution via multivariate curve resolution (MCR). Comparison with biochemical spectra of purified reference chemicals

We applied MCR to the Raman spectroscopic images of both native and engineered cartilage. We found that the MCR modeling produced degenerate collagen components (associated with collagen orientation) and these were summed to a single collagen component. Figure 2 displays the three biochemical pure spectra essentially representing collagen, GAG, and water, and accounting for 94.65% (collagen: 82.34%; GAG: 4.76%; water: 7.55%) of the model fit. The MCR resolved spectra for biochemical constituents correlated well with the laboratory grade pure biochemical, as evident by high correlation coefficients (*R*^2^ values of 0.84, 0.65, and 0.72 for GAG, collagen, and water in native cartilage, and values of 0.81, 0.61, and 0.89 for GAG, collagen, and water in engineered cartilage).

### Raman spectroscopic measurements of articular cartilage

**Fig. 3:**
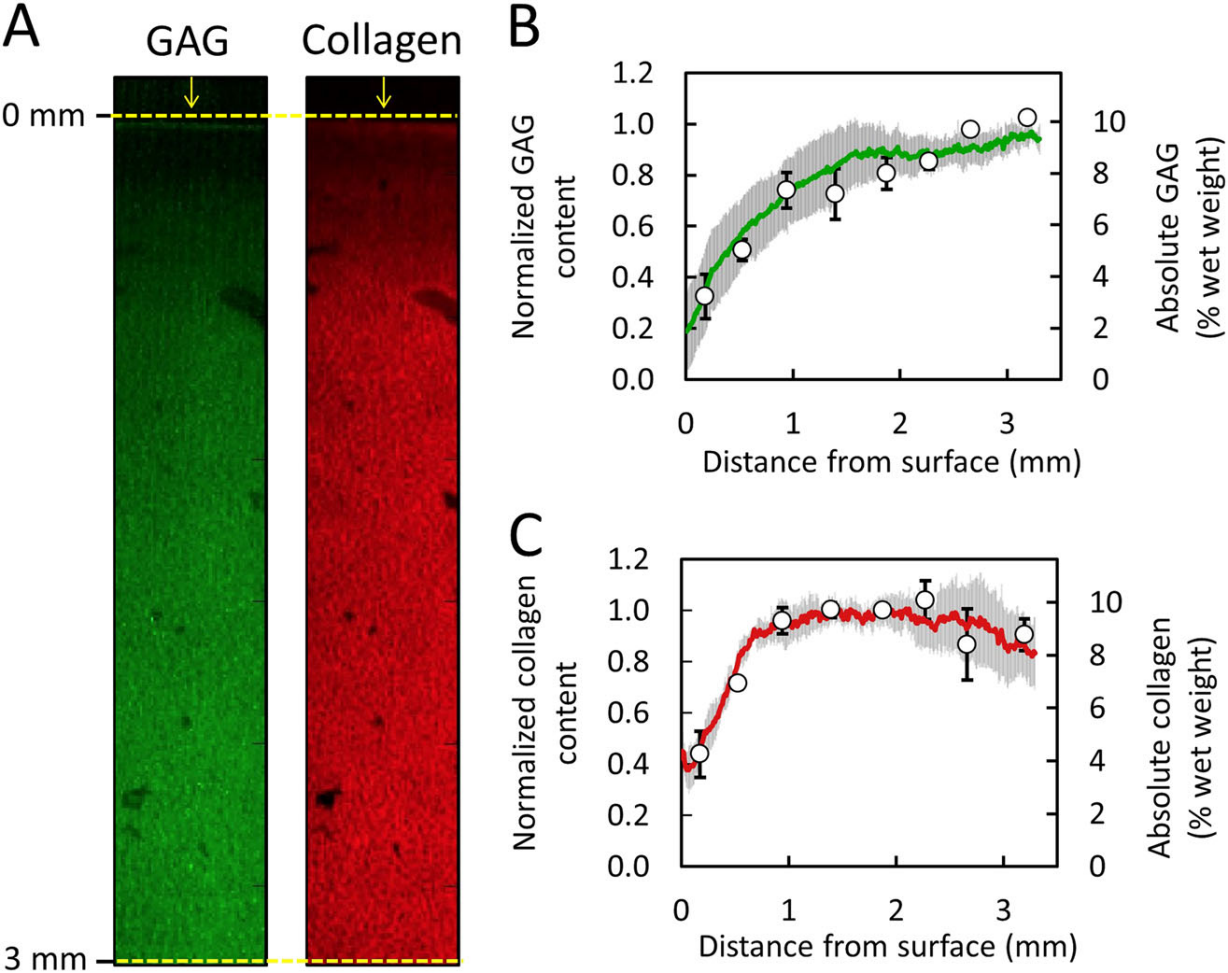
Validation of Raman spectroscopic imaging for semi-quantification of GAG and collagen concentration distribution in native cartilage. (**a**) Representative Raman spectroscopic images of GAG and collagen depth dependent concentration distributions from articular surface. Arrows represent articular surface. Agreement in depth dependent distribution profile of (**b**) GAG and (**c**) collagen as measured by Raman spectroscopic imaging (solid lines) and biochemical assaying (circle data points) (mean ± standard deviation)

Raman spectroscopic images of native articular cartilage exhibited depth dependent spatial gradients for GAG and collagen (Fig. 3a), whereby concentrations increased with distance from the articular surface. Biochemical assay measurements demonstrated similar depth dependent gradients: GAG increased from 3.3 ± 0.8% per wet weight near the articular surface (0.2 mm deep) to 10.3 ± 0.6% per wet weight at the deepest tissue level (3.2 mm deep), while collagen increased from 4.1 ± 0.1% to 8.2 ± 1.2% per wet weight over the same region (Fig. 3b–c). Following our data normalization scheme (described in Methods), biochemical and Raman distribution measures exhibited strong statistical agreement for GAG and collagen (Fig. 3b, c; *p* = 0.81, *R*^2  ^= 0.88 ± 0.05, RMSEP = 19.3% for GAG; *p* = 0.59, *R*^2  ^= 0.85 ± 0.13, RMSEP = 13.4% for collagen).

**Fig. 4:**
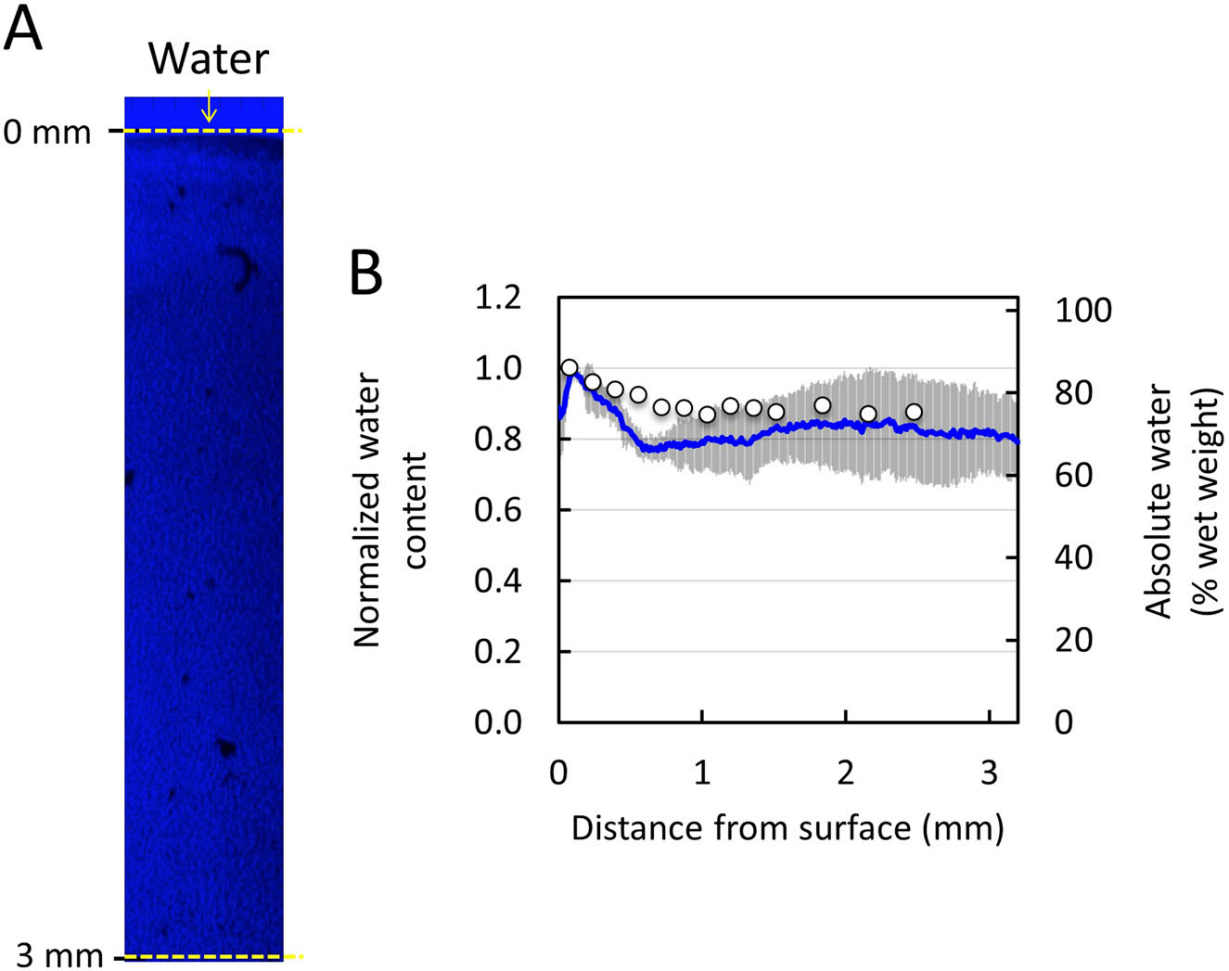
Validation of Raman spectroscopic imaging for semi-quantification of water concentration distribution in native cartilage. (**a**) Representative Raman spectroscopic image of water depth dependent concentration distributions from articular surface using high wave (~3400 cm^−1^) Raman signal. Arrows represent articular surface. (**b**) Agreement in depth dependent distribution profile of water as measured by Raman spectroscopic imaging (solid lines) and direct biochemical measurements from Oswald et al.^25^ (circle data points)

Raman spectroscopic images generated via the high wave number water signal (~3400 cm^−1^), yielded a depth dependent water gradient (Fig. 4a), whereby the concentration decreased with distance from the articular surface. According to Raman spectroscopic profile measurements, the relative water content decreased by 12.5% from the articular surface (0.1 mm deep) to the deepest tissue zone (2.5 mm deep), similar to measurements obtained by Oswald et al.,^25^ which demonstrated a relative decrease of 12% over the same tissue regions (Fig. 4b; *R*^2  ^= 0.64, RMSEP = 8.1%).

### Raman spectroscopic measurements of engineered cartilage

**Fig. 5:**
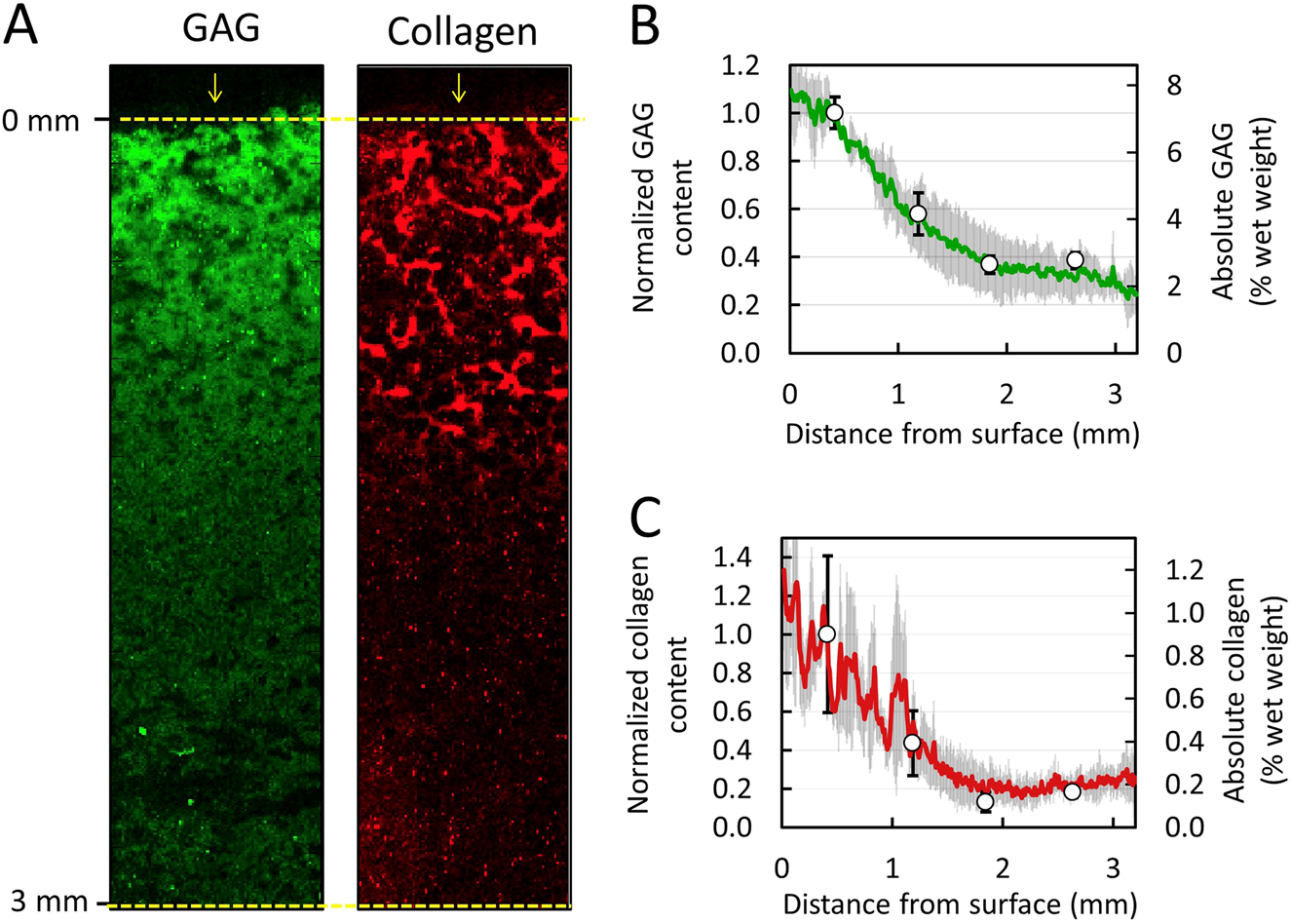
Validation of Raman spectroscopic imaging for semi-quantification of GAG and collagen concentration distribution in engineered cartilage constructs. (**a**) Representative Raman spectroscopic images of GAG and collagen depth dependent concentration distributions from media-exposed surface. Arrows represent media-exposed surface. Agreement in depth dependent distribution profile of (**b**) GAG and (**c**) collagen as measured by Raman spectroscopic imaging (solid lines) and biochemical assaying (circle data points) (mean ± standard deviation)

Raman spectroscopic images of engineered cartilage exhibited depth dependent spatial gradients for GAG and collagen (Fig. 5a), whereby concentrations decreased with distance from the media-exposed surface. Biochemical assay measurements demonstrated similar depth dependent gradients: GAG decreased from 7.3 ± 0.5% per wet weight near the articular surface (0.4 mm deep) to 2.8 ± 0.2% per wet weight at the deepest tissue level (2.6 mm deep), while collagen decreased from 0.9 ± 0.4% to 0.2 ± 0.1% per wet weight over the same region (Fig. 5b–c). Following data normalization, biochemical and Raman distribution measures exhibited strong statistical agreement for both constituents (Fig. 5b–c; *p *= 0.51, *R*^2  ^= 0.99, RMSEP = 6.3% for GAG; *p* = 0.22, *R*^2^ = 0.96, RMSEP = 10.5% for collagen).

### Raman spectroscopic measurements of digested cartilage

**Fig. 6:**
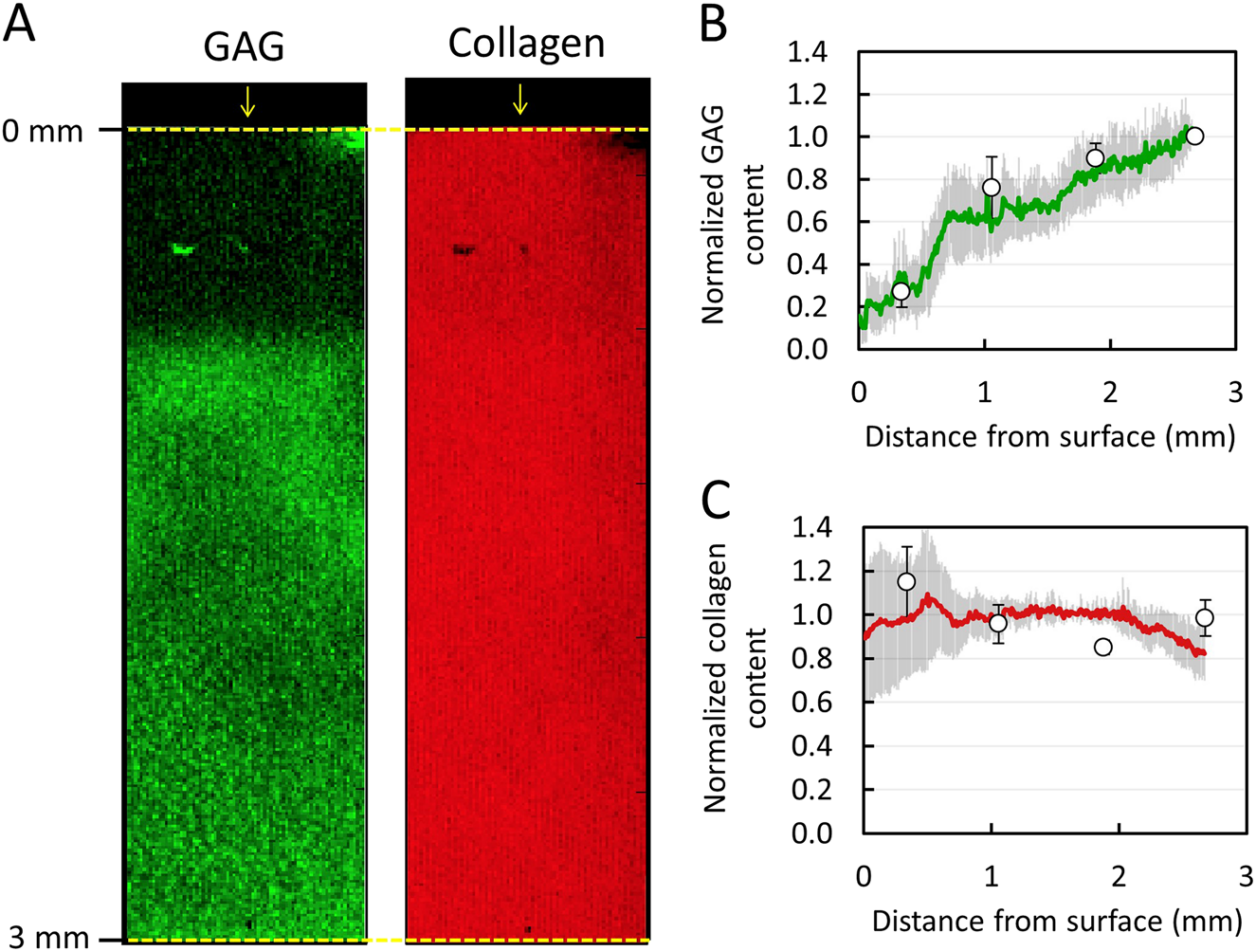
Validation of Raman spectroscopic imaging for semi-quantification of GAG and collagen distribution in enzymatically digested cartilage explant. (**a**) Representative Raman spectroscopic images of GAG and collagen depth dependent concentration distributions from trypsin-exposed surface. Arrows represent trypsin-exposed surface. Agreement in depth dependent distribution profile of (**b**) GAG and (**c**) collagen as measured by Raman spectroscopic imaging (solid lines) and biochemical assaying (circle data points) (mean ± standard deviation)

Raman spectroscopic images of enzyme treated cartilage explants exhibited a depth dependent GAG gradient, whereby the concentration increased with distance from the trypsin-exposed explant surface (Fig. 6a). The distribution of collagen remained near constant. Biochemical assay measurements demonstrated similar ECM constituent distributions; GAG increased from 1.7 ± 0.2% per wet weight near the articular surface (0.34 mm deep) to 6.7 ± 1.8% per wet weight at the deepest tissue level (2.7 mm deep), while collagen remained statistically uniform (*p* = 0.71) at a mean value of 9.7 ± 1.7% per wet weight (Fig S2B). Following data normalization, biochemical and Raman distribution measures exhibited strong statistical agreement for both constituents (Fig. 6b–c; *p* = 0.45, *R*^2^ = 0.93, RMSEP = 11.5% for GAG; *p* = 0.95, *R*^2^ = 0.83, RMSEP = 12.4% for collagen).

### Raman spectroscopy reveals localized heterogeneity in native and engineered cartilage

**Fig. 7:**
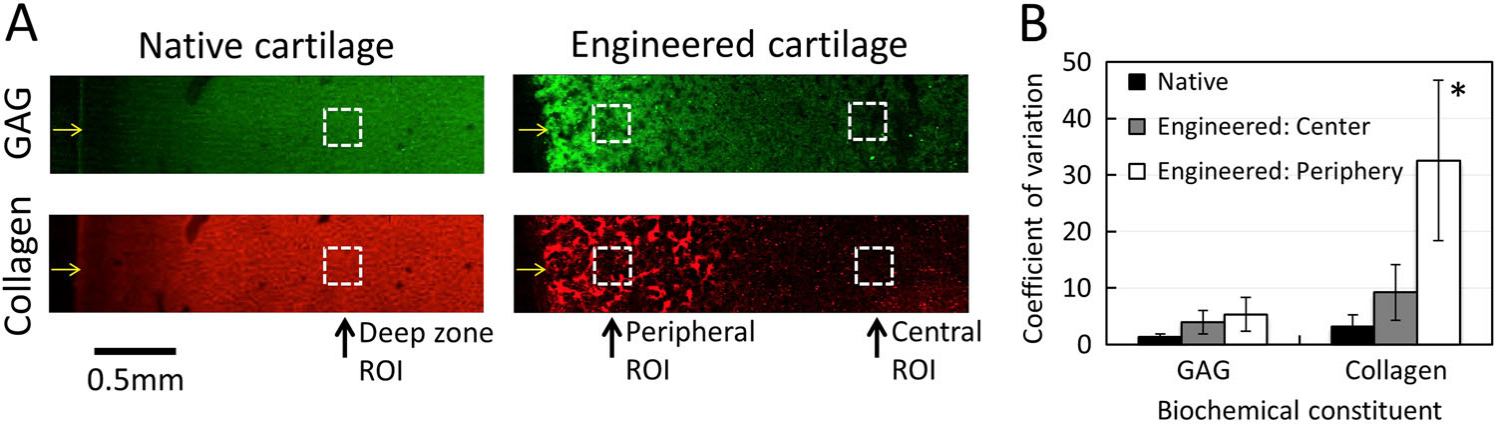
Raman spectroscopic measured localized heterogeneities in native and engineered articular cartilage. (**a**) Regions of interest (250 × 250 μm; dashed squares) for localized heterogeneity measurements from Raman spectral images of native and engineered cartilage tissues. Arrows represent tissue surfaces. The panel was reproduced from Fig. 3a and Fig. 5a (**b**) Localized heterogeneity in tissue regions as assessed by coefficient of variation measurements of Raman spectroscopic imaging data (mean ± standard deviation). *p < 0.05 represents a significant increase above corresponding native cartilage coefficient of variation

Native articular cartilage exhibited a low degree of localized tissue heterogeneity for both GAG and collagen with respective coefficient of variation values of 1.4 ± 0.5 and 3.2 ± 2.1 (Fig. 7). Engineered cartilage generally exhibited higher levels of localized heterogeneity; coefficients of variation were higher in the central region of engineered cartilage (GAG: 4.0 ± 2.1; collagen: 9.2 ± 4.9) and increased further in the engineered cartilage periphery (GAG: 5.4 ± 3.0; collagen: 32.6 ± 14.2).

## Discussion

The results of this study present the novel demonstration that Raman spectroscopic imaging can serve as an important analytical tool, providing highly accurate, high spatial resolution measurements of the distribution of cartilage ECM constituents in native and engineered cartilage tissues. A principal challenge in Raman spectroscopic imaging is the isolation of the signals of individual components with highly overlapping spectral patterns,^18^ such as the ECM constituents present in articular cartilage. Importantly, these results demonstrate that, following the acquisition of a Raman spectroscopic image of cartilage tissue samples, the implementation of MCR can successfully decompose and isolate the component spectra of the major ECM constituents of the tissue (GAG, collagen, and water), as evidenced by their high correlations with purified standard molecules (Fig. 2). It is important to note that the purified standard molecules are similar yet molecularly distinct from those present in the cartilage ECM, which experience a myriad of in situ chemical interactions, (e.g., fibril incorporation, crosslinking interactions), leading to the induction of protein conformational changes and additional molecular alterations of constituents. As such, the high correlation coefficients serve as a strong support for the spectral isolation and identification of ECM constituents in the tissue.

Based on the assumption that the Raman spectral intensity for each constituent is proportional to its concentration in the tissue, the successful implementation of this spectral decomposition, in principle, gives rise to the relative concentration of each constituent at each imaged pixel in the cartilage tissue sample. Since we use a confocal setup, optical properties of the tissue (absorption, scattering and anisotropy) have little influence on the detected signal and a linear model could be used. In order to assess the accuracy of these measurements, a novel validation technique was employed, whereby Raman concentration distribution measurements were compared to direct biochemical measurements at several discrete positions in the tissue. Results demonstrated exceptional agreement between Raman spectroscopic and biochemical assay measurements, as demonstrated by high correlation coefficients and low root mean square error of prediction values for both GAG and collagen (Table 1). This indicates that the intensities of their component spectra are indeed linearly proportional to their concentration in the tissue. As such, Raman spectroscopic imaging in conjunction with MCR is able to provide highly accurate measures of the relative distribution of these ECM constituents in cartilage tissues.

**Table 1:** Correlation coefficient (*R*^2^) and root mean square error of prediction (RMSEP) for tissue/constituent models between values obtained via Raman spectroscopic and biochemical assay measurements

Tissue model	Constituent	*R* ^2^	RMSEP [%]
Native cartilage	GAG	0.88	19.3
	Collagen	0.83	13.4
	Water	0.64	8.1
Engineered cartilage	GAG	0.99	6.3
	Collagen	0.99	10.5
Digested cartilage	GAG	0.93	11.5
	Collagen	0.83	12.4

The validation of the technique was successful for both native (Fig. 3) and engineered cartilage (Fig. 5), highlighting the broad applicability of this technique for cartilage-based tissues. Further, the ability of Raman imaging to accurately measure the biochemical distribution in GAG-digested cartilage (Fig. 6) serves as a critical demonstration that Raman spectroscopic imaging can faithfully distinguish between ECM constituents and successfully measure their concentration distributions in the tissue. Given the validation of this study, Raman spectroscopic imaging can now be utilized as a robust analytical tool for assessing the distribution of the ECM in cartilage-based tissue samples. It is important to note that, by itself, Raman spectroscopic imaging provides measures of the semi-quantitative or relative distribution of ECM constituents in cartilage tissues. As performed in this study (Figs. 3–5), the relative distribution can readily undergo conversion to a fully quantitative absolute concentration distribution, provided knowledge of the average constituent concentration in the imaged sample. This average value can be readily obtained via a single biochemical assay measurement, performed on either the Raman imaged sample (subsequent to imaging) or on a lateral symmetric tissue region (as performed in Fig. 3). However, while not performed in this study, we anticipate that in the future, Raman spectroscopic imaging can be used to measure the absolute concentration distribution of ECM constituents through the use of systems with well controlled laser excitations or the introduction of molecular standard molecules with unique Raman spectral peaks in the tissue.

A promising advantage of Raman spectroscopic imaging is the ability to perform measurements on hydrated specimens, giving this technique the unique potential of being able to characterize the distribution of water in biological tissues. To this end, results demonstrated strong agreement between Raman spectroscopic and direct biochemical measurements of water in native cartilage tissues (Fig. 4). Not surprisingly, the high-wavenumber spectral range 3100–3600 cm^−1^ provided a more accurate estimation of the water content compared to the fingerprint range (800–1800 cm^−1^) (Fig S3). This is most likely because the signal intensity (sensitivity) of water is inherently low in the fingerprint spectral range. On the other hand, due to the high biomolecular specificity, the fingerprint range proved excellent to resolve collagen from GAGs. This demonstration represents one of the first high resolution water distribution measurements in articular cartilage. We anticipate that the small deviation between Raman spectroscopic and biochemical measured water content in the tissue middle zone (~0.6–1.5 mm deep) could potentially be attributed to a disparity in different pools of water measured by the two techniques. The biochemical measurements (tissue wet weight to total weight ratio) performed by Oswald et al.^25^ predominantly reflect the free (unbound) water in the tissue. Alternatively, the Raman spectroscopic water measurements performed in this study likely represent a combination of the pools of free and bound water, as observed previously in bone^26^ and cartilage specimens.^27^ In future work, we anticipate being able to distinguish between these pools via additional analysis. We must note that water distribution measurements in engineered cartilage specimens were not performed in this current investigation due to an excessive background signal of these tissues. However, in the future, we anticipate that this issue can readily be remedied through use of near-infrared laser light excitation.

While a host of ECM distribution assessment techniques have been previously utilized for cartilage, they are often associated with limitations in regards to molecular specificity, spatial resolution and accuracy. The most commonly utilized ECM distribution analytical technique, histochemical staining, involves sample processing techniques that are difficult to standardize and are often associated with a variety of measurement artifacts.^8^ Notable issues include the loss of ECM during sample fixation,^28,29^ a lack of specificity of visualization dyes,^30,31^ and non-stoichiometric dye binding.^32,33^ Consequently, histochemical staining is conventionally viewed as a qualitative assessment tool for cartilage ECM distribution analysis. While more robust analytical techniques for cartilage ECM measurements have recently emerged, such as Fourier transform infrared spectroscopy imaging, they are also associated with critical limitations, such as a low spatial resolution (~5 μm^34^) and a lack of compatibility with unfixed, hydrated tissue specimens. As such, Raman spectroscopic imaging possesses considerable benefits above prior tissue characterization techniques, including quantitative accuracy, diffraction limited spatial resolution, and compatibility with hydrated tissue specimens.

Raman spectroscopic imaging now has the potential to serve as a highly valuable tool for articular cartilage analysis, allowing for novel semi-quantitative assessments of the distribution of ECM in the tissue. Importantly, results of our study demonstrate that this technique has the ability to measure cartilage ECM distributions on multiple length scales, including throughout the entire tissue depth (via profile averaging) (Figs. 3 and 5) and in localized regions (via coefficient of variation calculations) (Fig. 7). In one important application of this technique, Raman spectroscopic imaging can be used to provide novel, improved characterizations of cartilage degeneration during osteoarthritis. During the onset and progression of osteoarthritis, the organization of the cartilage ECM is disrupted, in turn compromising the mechanical functionality of the tissue. To this end, Raman spectroscopic imaging can be used to precisely and accurately measure changes to the distribution of ECM, leading to an improved understanding of the process by which cartilage degenerates during disease progression. Raman spectroscopic imaging distribution measurements can readily be acquired on a range of length scales, allowing for characterizations of both the tissue level (e.g., depth dependent ECM distributions) and cellular level (e.g., pericellular matrix to extracellular matrix transitions). Furthermore, while the current study focuses on the identification of GAG, collagen, and water levels in tissues, we anticipate that future models may be able to achieve increased biomolecular specificity and potentially distinguish between types of collagen (collagen-I, collagen-II, collagen X), GAGs (e.g., heparin, chondroitin sulfate), and water (free, intrafibrillar).^35^

**Fig. 8:**
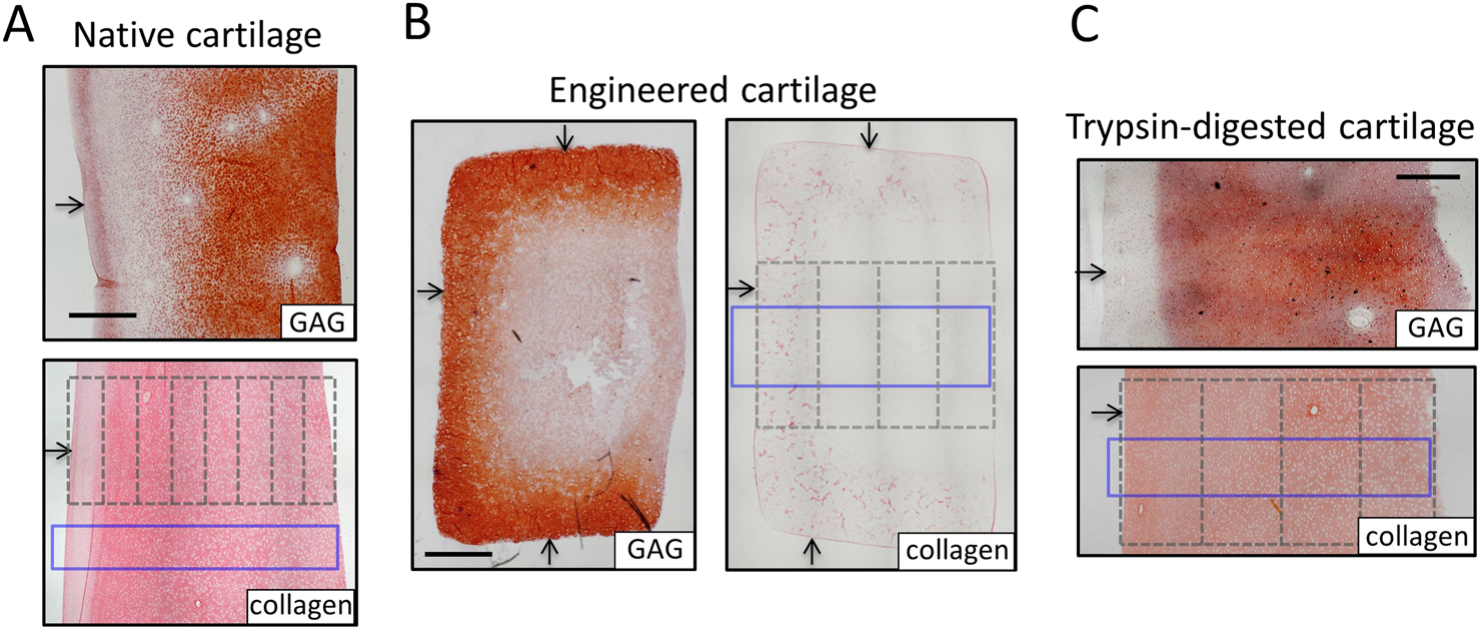
Tissue models for Raman spectroscopic measurement validations. Histological staining of ECM constituent spatial gradients (Safranin-O [GAG] and Picrosirius Red [collagen]) for tissue sections. (**a**) Native bovine articular cartilage possesses GAG and collagen distributions that increase from the articular surface; the distribution of water decreases from the surface (not shown). (**b**) Large engineered cartilage tissue constructs (∅6 × 3 mm) possess GAG and collagen distributions that decrease from the media-exposed surface. (**c**) Trypsin-exposed deep zone bovine cartilage possesses a GAG distribution that decreases from the trypsin-exposed surface and a near-uniform collagen distribution. Arrows represent articular surface, media-exposed surfaces, and trypsin-exposed surface for each respective tissue type. Dashed lines represent outline of tissue sections for biochemical assaying. Solid blue rectangle represents region of interest for Raman spectroscopic imaging. Scale bars represent 1 mm

In a second application, Raman spectroscopic imaging can be applied as a valuable analytical tool for cartilage tissue engineering strategies. A major goal of cartilage tissue engineering is to generate replacement tissues that recapitulate the native cartilage ECM organization. Interestingly, in recent years, a variety of tissue engineering strategies have been particularly focused on the development techniques to improve spatial control of the distribution of ECM deposition during tissue growth. For example, as illustrated in the tissue growth model of this study (Fig. 8b), it has recently been shown that the conventional strategy of supplementing culture medium with growth factors can give rise to undesirable heterogeneities in ECM^36–38^: (1) depth dependent heterogeneities that result from growth factor transport limitations and (2) localized heterogeneities induced by excessive growth factor activity at the tissue periphery. A series of studies have attempted to develop novel growth factor delivery strategies (such as nutrient channel implementation, scaffold-growth factor conjugation) in order to mitigate ECM heterogeneities.^38–40^ To this end, Raman spectroscopic imaging can be used to assess the degree of both depth dependent and local heterogeneities in engineered tissues and, as such, be used to assess the success of tissue growth strategies. In another example, other strategies have been implemented in attempts to recapitulate the zonal depth dependent native ECM organization in engineered tissues.^41–44^ Raman spectroscopic imaging can be used to measure the distribution of ECM in engineered cartilage in order to gauge the success of zonal organization development.

 This study serves as a powerful validation of the accuracy of Raman spectroscopic imaging measurements of the spatial distribution of biochemical components in a biological tissue. This validation has exceptionally broad applicability, serving as strong support for the reliability of biochemical spatial distribution measurements acquired from Raman spectroscopic images for an expansive variety of tissues. In particular, these validations on cartilage tissues offer the high likelihood that Raman spectroscopic images can produce highly accurate ECM distribution measurements for a host of additional musculoskeletal tissues (e.g., tendon, meniscus, and intervertebral disk) due to their ECMs that are structurally similar to articular cartilage and made up of similar biochemical components. As such, Raman spectroscopic imaging can become a powerful analytical tool routinely implemented for musculoskeletal tissue research.

## Methods

### Tissue analyses

#### Native articular cartilage

Immature articular cartilage explants were harvested from the femoral condyles of 4-week-old calves (*N* animals) procured from a local slaughterhouse. All animal tissue procurement protocols were performed with institutional approval from Imperial College. A portion of the explant deep zone was excised, creating cylindrical explants of ∅6 × 3.5 mm dimensions with the articular surface intact. Explants were stored at −30 °C for up to 2 weeks until testing. These native explants possess concentration gradients of GAG and collagen, whereby levels increase from the articular surface towards the deeper region of the tissue (Fig. 8a).

The concentrations of GAG and collagen were measured in sectioned slices of cartilage explants (n = *4*; one explant per animal) via biochemical assaying (as described in Biochemical Assay Measurement Methodology), yielding the concentration of these ECM constituents as a function of distance or depth from the articular surface. The depth dependent distribution of the water content was adopted from Oswald et al.,^25^ where measurements were performed on the same tissue type. In order to allow for statistical comparison with Raman spectroscopic measurements, measured ECM constituent concentrations were normalized using the following scheme to yield the relative concentration distribution. For constituents with increasing depth dependent concentrations (GAG and collagen), concentrations were normalized to the peak concentration value (depth position 1.4 mm). For constituents with decreasing depth dependent concentrations (water), concentrations were normalized to the concentration in the topmost biochemical section (depth position 0.1 mm).

Raman spectroscopic distribution images of ECM constituents were acquired (as described in Raman Spectroscopy Methodology) in a lateral region of the same cartilage explants (Fig. 8a), allowing for a pair-wise statistical comparison of the distribution between the two techniques. Preliminary measurements show that the biochemical content of native cartilage exhibits minimal variation in the lateral direction, thus supporting the use of this paired analysis (Fig S1). For each molecular constituent (GAG, collagen, and water), pixel intensities were averaged across the lateral region of interest dimension, yielding its depth dependent relative concentration distribution in arbitrary units. These intensity distributions were normalized using the same positional values used for biochemical assay normalization (described above); this allowed for a statistical comparison of the relative concentration distribution between Raman spectroscopic and direct biochemical measurements. Additionally, for illustration purposes, normalized Raman spectroscopic measured concentrations were converted to absolute concentrations by multiplying the Raman concentration distribution by the average concentration in all biochemical measured tissue specimens.

Raman spectroscopic images were also used to quantitatively measure the degree of localized heterogeneity in different regions of cartilage tissues. Here, in native cartilage tissues, measurements were performed by calculating the coefficient of variation of pixel concentrations of GAG and collagen within a localized (250 × 250 μm) region of interest in the tissue deep zone.

#### Engineered cartilage

Immature primary articular chondrocytes were isolated from the carpometacarpal joints of 4-week-old calves (*N* = 4 animals) and encapsulated in 2% w/v type VII agarose at a nominal density of 50 × 10^6^ cells/mL, as described previously.^45^ Large tissue constructs (∅6 × 3.2 mm) were cultured for seven weeks in a chondrogenic media formulation, consisting of high glucose DMEM supplemented with 100 nM dexamethasone, 100 μg/mL sodium pyruvate, 50 μg/mL L-proline, 1% ITS + premix (6.25 μg/mL insulin, 6.25 μg/mL human holotransferrin, 6.25 ng/mL selenium), 1% PS/AM antibiotic antimycotic, and 173 μM ascorbic acid 2-phosphate. TGF-β3 was supplemented in media at 10 ng/mL for the initial 2 weeks of culture. These engineered cartilage constructs possess concentration gradients of GAG and collagen, whereby levels decrease from the media exposed construct surface towards the construct center (Fig. 8b), as shown previously.^38^

The concentrations of GAG and collagen were measured in sectioned slices of a cylindrical sub-cored engineered tissue construct (*n* = 6) via biochemical assaying, yielding the concentration of these ECM constituents as a function of distance or depth from the media-exposed tissue surface (Fig. 8b). For both constituents, concentrations were normalized to the concentration in the topmost biochemical section (depth position 0.4 mm).

Raman spectroscopic distribution images of ECM constituents were acquired from additional tissue constructs (*n* = 4) over a central region of interest (Fig. 8b). For GAG and collagen, pixel intensities were laterally averaged, yielding their depth dependent relative concentration distributions. Distributions were normalized to the concentration at the 0.4 mm depth position for statistical comparison with biochemical assay measurements.

The degree of localized heterogeneity was further measured for engineered cartilage tissues. Here, the coefficient of variation of pixel concentrations of GAG and collagen (within a localized 250 × 250 μm region of interest) was calculated at both the tissue peripheral region (~100 μm from the media exposed surface) and central region (~2 mm from the media exposed surface).

#### Digested articular cartilage

Native articular cartilage explants possess a similar biochemical gradient for each ECM constituent (GAG and collagen), marked by a monotonic concentration increase through the depth of the tissue (Fig. 8a). In an effort to better validate the ability of Raman spectroscopic imaging to quantitatively distinguish between levels of GAG and collagen in cartilage tissues, an additional analysis was performed on samples that possess disparate gradients of GAG and collagen. In order to generate cartilage with disparate gradients, the following protocol was adopted. Here, the superficial and middle zones (topmost 1 mm) of cartilage explants (from 4-week-old calves) were excised, yielding ∅6 × 2.5 mm deep zone explants that possess near uniform distributions of both GAG and collagen. Subsequently, the explants were exposed to trypsin, an enzyme that predominantly digests and extracts GAG from the tissue while maintaining collagen levels unaltered (Fig S2). Specifically, explants were affixed via cyanoacrylate glue to the bottom surface of polystyrene well plates and exposed to trypsin (50 μg/mL in PBS) for 10 h at 37 °C. Following digestion, ∅3 mm axial cylindrical subcores were extracted from the center of each digested explant, yielding cartilage disks that possess a profile of increasing GAG concentration throughout their depth but maintain a uniform collagen distribution (Fig. 8c and Fig S2).

The concentration of GAG and collagen were measured in sectioned slices of the ∅3 mm sub-core tissue (*n* = 4; one explant per animal) via biochemical assaying, yielding the concentrations of these constituents as a function of distance or depth from the trypsin-exposed tissue surface. For GAG, concentrations were normalized to the peak concentration value (depth position 2.5 mm). Since collagen levels did not significantly vary with depth, concentrations were normalized to the average concentration in all section slices.

Raman spectroscopic distribution images were acquired from additional trypsin treated explants (*n* = 4; one explant per animal) in the depicted region of interest (Fig. 8c). For GAG and collagen, pixel intensities were laterally averaged, yielding their depth dependent relative concentration distribution. Distributions were normalized (to the concentration at the 2.5 mm depth position for GAG; average value for collagen) for statistical comparison with biochemical assay measurements.

### Biochemical tissue assessment

#### Biochemical assay measurements

Cylindrical explants of articular and engineered cartilage were analyzed for their depth dependent biochemical distribution, as described previously.^38^ Here, ∅3 mm axial cylindrical subcores were extracted throughout the depth of each tissue and then transversely cut into thin depth-dependent tissue sections (eight sections for native cartilage; four sections for engineered constructs and digested cartilage; Fig. 8). Each section was digested via proteinase-K (0.5 mg/mL) and subsequently processed for its GAG and collagen contents, via the dimethylmethaline blue^46^ and orthohydroxyproline^47^ assays, respectively. Biochemical contents were normalized by tissue wet weights, yielding a depth-dependent biochemical concentration distribution in the tissue from the sample’s top surface (articular surface for native explants, media exposed top surface for engineered constructs, and trypsin exposed surface for digested cartilage).

#### Histology

Native and engineered cartilage tissues were fixed overnight in 3.7% paraformaldehyde, 5% acetic acid, and 70% ethanol, paraffin embedded, and sectioned. Sections were stained for GAG and collagen visualization with 1% safranin-O and 0.1% picrosirius red, respectively, while counterstained with hematoxylin.

### Raman spectroscopic imaging

#### Sample preparation

The successful acquisition of Raman spectroscopic images requires the utilization of highly flat tissue specimens to ensure that the laser focal plane does not drift throughout the tissue. To this end, special care was taken to ensure that specimens imaged in this study were flat and aligned with the Raman microscope stage. Here, the imaging and under surface of native and engineered cartilage tissues were trimmed to obtain flat parallel surfaces. Native tissues were trimmed using a cryostat microtome [Bright Instruments OTF 5000] and the relatively softer engineered cartilage samples were trimmed with a vibrating microtome [Campden Instruments Model 7000]. Both tissue types were anchored onto a flat polystyrene square using a cyanoacrylate adhesive (applied outside the imaging field of view) and stored at 4 °C in PBS until Raman spectroscopic imaging was performed. Further, to avoid a potential bias in measurements from the direction of tissue scanning, sample orientation on the translational stage was altered for each sample.

#### Raman spectroscopic instrumentation

We applied a confocal Raman microspectroscopy system (Alpha 3000, Witec, GmbH.). The microscope stage was equipped with a piezoelectric stage (UHTS 300, Witec, GmbH.). A green laser (*λ*_ex_ = 532 nm, Witec, GmbH.) with a maximum output of 75 mW was fiber-coupled into the microscope using a 10 µm single mode silica fiber. Raman images were measured using a Leica 10 × /0.25 water immersion objective. The backscattered Raman photons were fed into an imaging spectrograph (UHTS 300, Witec, GmbH.) with a 600 groove/mm grating using a 100 μm silica fiber. The spectrometer was equipped with a thermoelectrically cooled (−60 °C), charge-coupled device camera (Newton, Andor Technology Ltd. UK). The confocal Raman system acquires spectra covering the range from 0 to 3600 cm^−1^. To calibrate the wavelength axis, the atomic emission lines of the argon/mercury spectral calibration lamp (HG-1, Ocean Optics, Inc.) were used.

#### Raman spectroscopic imaging of tissue samples

Raman images (∼3500 × 1000 μm (spatial resolution of ∼10 μm)) of native articular cartilage and tissue engineered constructs were measured by continuous scanning of the sample. Each Raman spectrum was collected with an acquisition time of ~0.5 s and a power on the sample of ∼41 mW using the 532 nm laser excitation. We did not observe any sample degradation using this power density.

#### Multivariate curve resolution

Before image analysis, the Raman spectra were pre-processed. To remove tissue autofluorescence, each spectrum was baseline subtracted using the shape function with a window width of 1000 data points using Project FOUR software version 4.0 (Witec, GmbH) (Figure S4). All Raman spectroscopic images were then combined into a single dataset and analyzed together using non-negativity constrained MCR according to our recently developed image analysis framework.^48^ Briefly, MCR aims to deconvolve each pixel Raman spectrum into its pure components and associated abundances through the linear equation:

$${\mathrm{D}} = {\mathrm{CS}} + {\mathrm{E}}$$

where D is a matrix containing the raw spectra, C is the relative concentrations, S is the pure biochemical spectra, and E is the residuals. In MCR, this equation can be solved using the alternating least squares algorithm.^19^ In this work, a model complexity of four components was chosen that represented collagen, glycosaminoglycans, water, and a residual associated with chondrocytes. Due to the higher molecular specificity we fit the MCR model to the fingerprint range only (800–1800 cm^−1^). To further validate that the system was sensitive to the respective components, we correlated the extracted pure biochemical spectra with those of laboratory grade reference biochemicals (Sigma Aldrich). Multivariate statistical analysis was conducted using PLS-toolbox (Eigenvector Research, Manson, WA) and in-house written scripts in the Matlab 2014a (Mathworks, Natick, MA) programing environment.

Since the water signal is inherently weak in the fingerprint range 800–1800 cm^−1^, we took advantage of the high-wavenumber range 2800–3600 cm^−1^ to quantify the tissue hydration.^49^ Here we use a univariate approach by mapping the water content using the broad band associated with OH stretching vibrations near ~3400 cm^−1^.

### Statistical analysis

Two-way ANOVAs were performed (*α* = 0.05 and statistical significance set at *p* < 0.05) to assess the effect of measurement technique (Raman spectroscopic imaging and biochemical assay) and depth on the ECM concentration (GAG or collagen) in native, engineered, or trypsin-digested cartilage. Tukey’s HSD post-hoc tests were run to examine differences between experimental groups.

The root mean square error of prediction (RMSEP) (as percentage of mean value of the data) between Raman spectroscopic and biochemical measurements was calculated for each tissue-constituent model, as performed in prior spectroscopic investigations.^50^

### Data availability

Raw data is available at 10.5281/zenodo.1115318.

## Electronic supplementary material

Supplementary Information
