# Supplementary material for: Raman spectroscopic imaging for quantification of depth-dependent and local heterogeneities in native and engineered cartilage
Source: NPJ Regen Med. 2018 Feb 9;3:3. doi: 10.1038/s41536-018-0042-7 (PMC5807411; doi:10.1038/s41536-018-0042-7)
Supplement: Supplementary file 1 — Supplementary Information [file 41536_2018_42_MOESM1_ESM.docx]

# Supplementary Information:

# Raman Spectroscopic Imaging for Quantification of Depth-Dependent and Local Heterogeneities in Native and Engineered Cartilage

Albro MB^†‡^*^§^*^a^, Bergholt MS^†‡^*^§^*^a^, St-Pierre JP^†‡^*^§^*, Vinals-Guitart A^†‡^*^§^*, Zlotnick H^†‡^*^§^*, Stevens MM^†‡^*^§*^*

^†^*Department of Materials, Imperial College London, London SW7 2AZ, United Kingdom*

^‡^*Department of Bioengineering, Imperial College London, London SW7 2AZ, United Kingdom*

*^§^Institute of Biomedical Engineering, Imperial College London, London SW7 2AZ, United Kingdom*

^a^Denotes equal contribution

***Correspondence:** Prof. Molly M. Stevens, E-mail: m.stevens@imperial.ac.uk.

**Running Title:** Raman Spectroscopy of Native and Engineered Cartilage

# Supplementary Figures

**
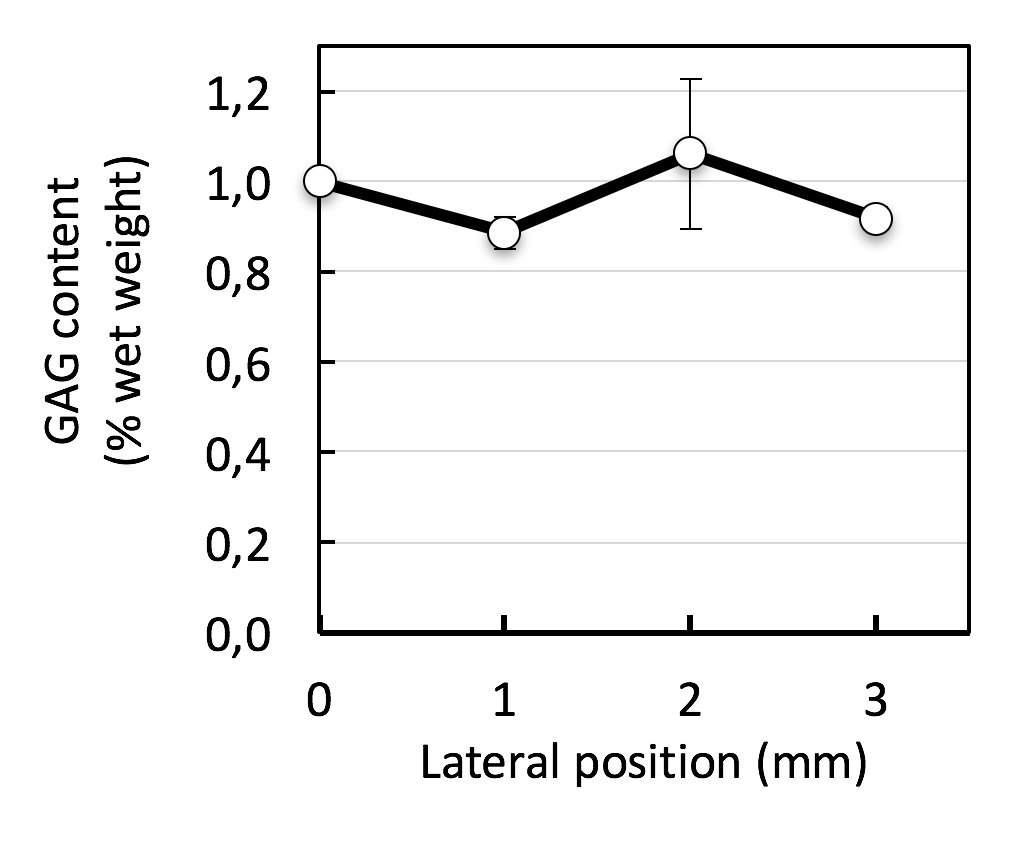
**

**Figure S1:** Glycosaminoglycan content of native cartilage exhibits minimal variation in the lateral direction. This demonstration supports the use of a statistical paired comparison between measurements made via Raman spectroscopic imaging and biochemical assaying on contiguous lateral native explants, as performed in the native cartilage validation (Fig 4).


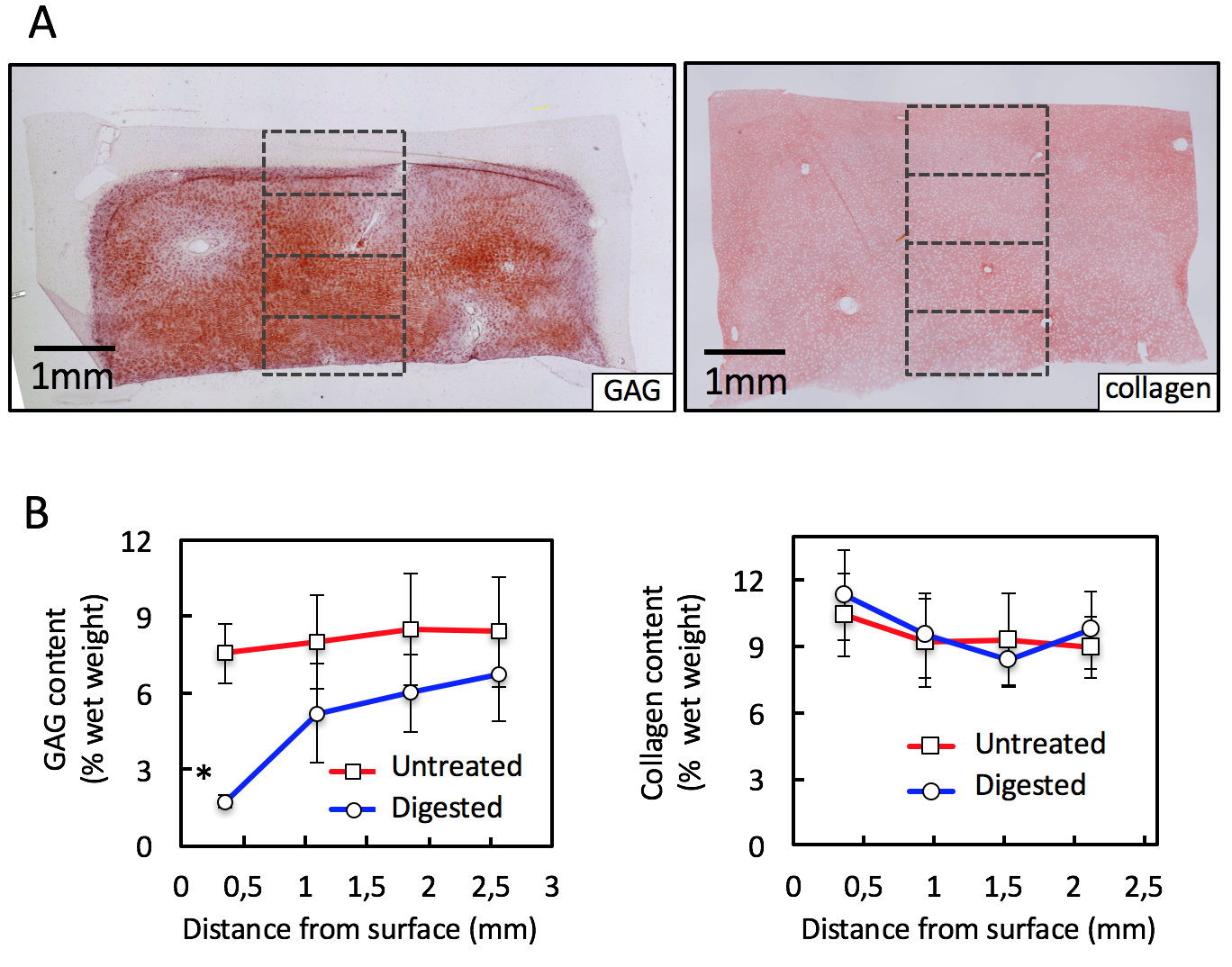


**Figure S2:** (A) Histological staining (Safranin-O [GAG] and Picrosirius Red [collagen]) and (B) spatial biochemical measurements in deep zone cartilage explants after 6 hour treatment with 50 μg/mL trypsin enzyme. Trypsin digestion induces depth dependent GAG gradients while maintaining a uniform distribution of collagen. Dashed lines represent outline of tissue sections for biochemical assaying. *p<0.05 represents statistical decrease below corresponding untreated control value.

**
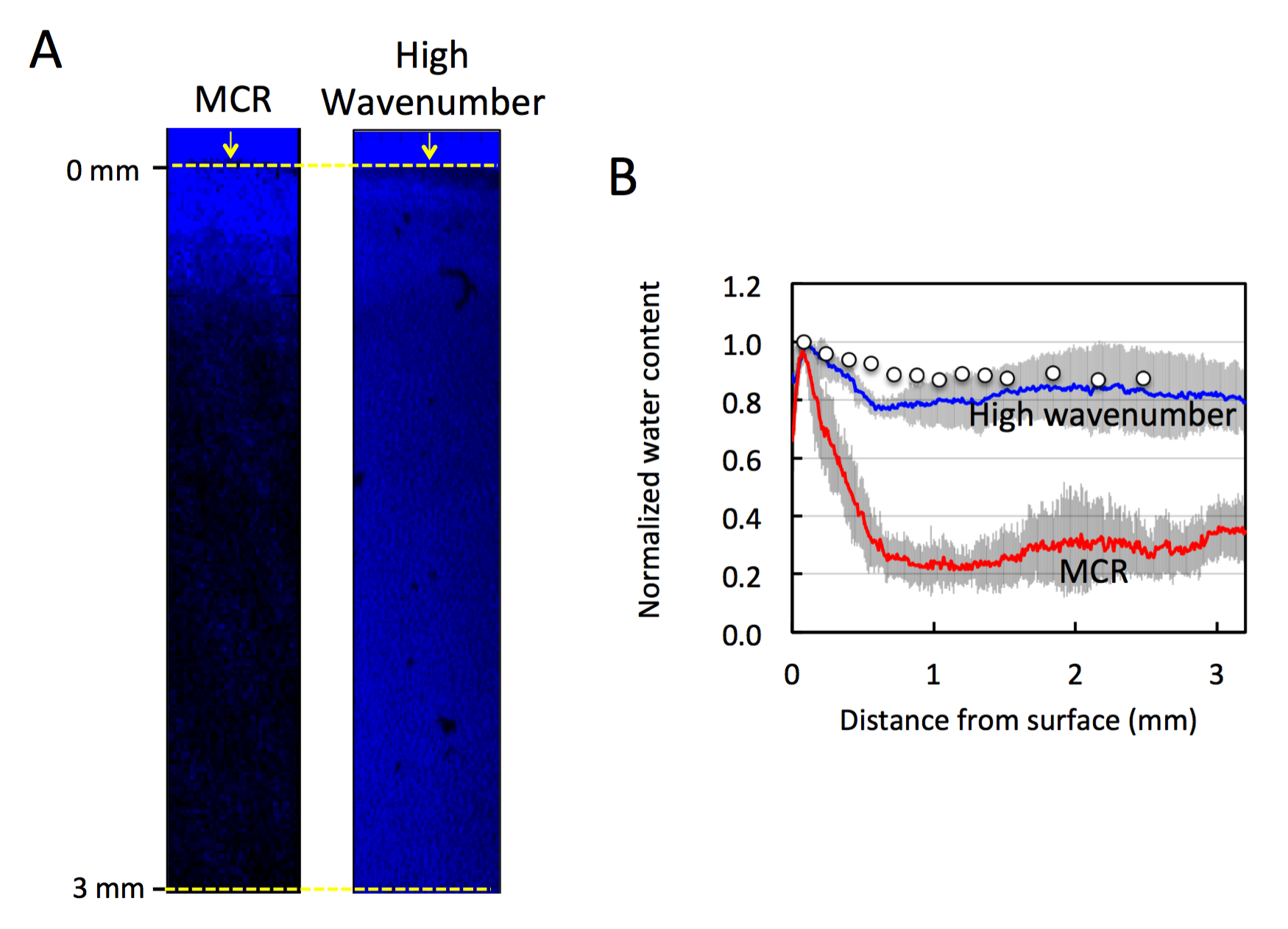
**

**Figure S3:** (A) Depth dependent concentration distribution of water acquired via high wavenumber peak (~3400 cm^-1^) and water component spectra following deconvolution via multivariate curve resolution (MCR) in the fingerprint range 800 - 1800 cm^-1^. (B) Depth dependent distribution profile of water as measured by Raman spectroscopic imaging (solid lines) and biochemical assaying (circle data points) from Oswald *et al.* [*^1^*](#_ENREF_1). Only distribution via high wavenumber peak agrees with direct biochemical measurements of water content.


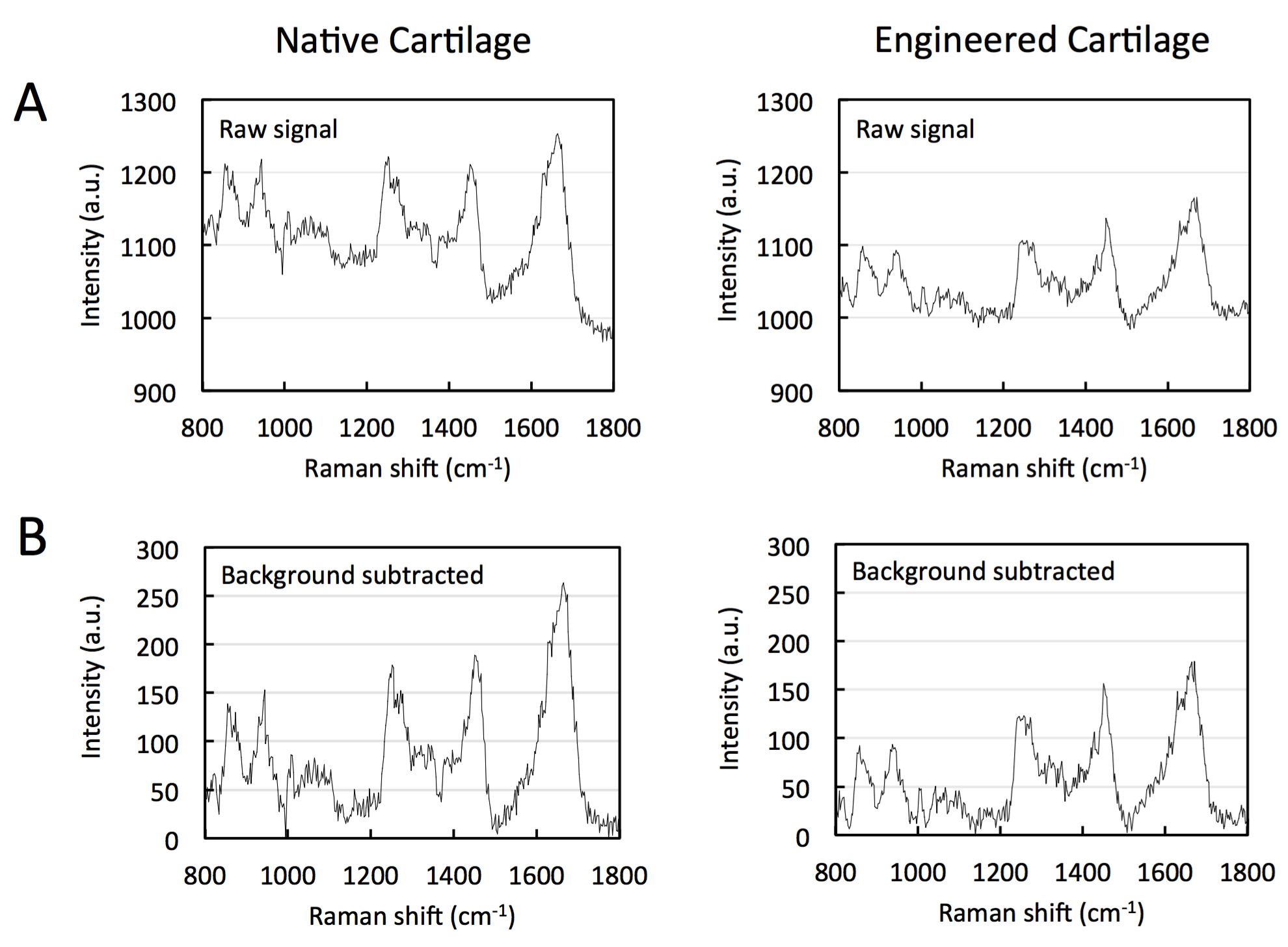


| **Figure S4**: (A) Representative raw spectra of native and engineered cartilage samples, and (B) spectra after background subtraction. |
| --- |

**Supplementary References**

1. Oswald, E.S., Chao, P.H., Bulinski, J.C., Ateshian, G.A. & Hung, C.T. Dependence of zonal chondrocyte water transport properties on osmotic environment. *Cellular and molecular bioengineering* **1**, 339-348 (2008).
